# Supplementary material for: Changes in the spectrum of kidney diseases: a 14-year single-center retrospective renal biopsy study from southeast China
Source: PeerJ. 2025 Apr 14;13:e19302. doi: 10.7717/peerj.19302 (PMC12005175; doi:10.7717/peerj.19302)
Supplement: Supplemental Information 2 [file peerj-13-19302-s002.docx]

Supplementary Table1. Classification of biopsy-proven renal disease

| **Primary Glomerulonephritis (PGN)** |
| --- |
| Membranous nephropathy (MN)  IgA nephropathy (IgAN)  Minimal change disease (MCD)  Focal segmental glomurular sclerosis (FSGS)  Mesangial proliferative glomerulonephritis (MsPGN)  Membranoproliferative glomerulonephritis (MPGN)  Endocapillary proliferative glomerulonephritis (EnPGN)  Sclerosing glomerulonephritis |
| **Secondary Glomerulonephritis (SGN)** |
| Lupus nephritis (LN)  Diabetic nephropathy (DN)  Hepatitis B-associated nephritis (HBVN)  Hypertensive nephropathy (HTN)  Henoch-Schönlein purpura nephritis (HSPN)  ANCA-associated glomerulonephritis  Anti-GBM glomerulonephritis  Infection-related glomerulonephritis  Renal amyloidosis  Monoclonalimmunoglobulin deposition disease  Obesity-related glomerulonephropathy  Thrombotic microangiopathy |
| **Tubulointerstitial Nephritis (TIN)** |
| Acute tubular necrosis  Acute interstitial nephritis  Chronic interstitial nephritis |
| **Other Renal Diseases** |
| Hereditary renal diseases  Alport syndrome  Fabry disease  Thin basement membrane syndrome  Drash syndrome  Rare kidney diseases  Radiation nephropathy  Lipoprotein nephropathy |
